# Improving compliance with physical distancing across religious cultures in Israel

**DOI:** 10.1186/s13584-021-00501-w

**Published:** 2021-11-24

**Authors:** Gillie Gabay, Attila Gere, Lior Naamati-Schneider, Howard Moskowitz, Mahdi Tarabieh

**Affiliations:** 1 Achva Academic College, 7980400 Arugot, Israel; 2 Postharvest Science and Sensory Evaluation, Szent István University, Budapest, Hungary; 3 Health Services Management, Hadassah Academic College, Jerusalem, Israel; 4 Mind-Genomics Associates, White Plains, NY USA; 5 Faculty of Nursing, Tel Aviv-Jaffa Academic College, Tel Aviv, Israel

**Keywords:** Compliance, COVID-19, Epidemiology, Health policy, Messaging, Physical distancing, Religious minorities

## Abstract

**Background:**

Physical distancing contains the corona virus, but compliance with physical distancing across religious minorities in Israel has been shown to be relatively poorer than in the majority population. This study tests the power of messages as drivers of willingness to comply with physical distancing across religious minorities in Israel during the first wave of the COVID-19 from March till June 2020.

**Methods:**

896 Israeli Muslims, Druze, Bedouins, Jewish Orthodox, Christians, and Jewish Seculars participated in this conjoint-based experimental design. The size of the total sample and of the subgroups is consistent with the suggested size in conjoint analysis studies, particularly when aiming at stability of coefficients rather than stability of means. The dependent variable was ‘willingness to comply’. Independent variables were known contributors to compliance: perceived risk, practices of physical distancing, ways to assure compliance, and the agent communicating the policy.

**Results:**

A regression analysis indicated minor differences in the power of messages across groups despite dramatic cultural differences amongst them. We identified three distinct mindsets that transcend religious cultures from the responses of the study subjects to various messages and named them “pandemic observers,” “obedient followers,” and “sensitive interpreters.” Compliance of "Pandemic Observers" (n = 306) may be improved by messages such as, “Dangerous virus spreading wildly” and “Health experts suggest what to do but the government is reactive rather than proactive” (β = 14, p < .005). Compliance of "Obedient Followers" (n = 242) may be driven by the messages “Socialize and work only from home, using the internet, e.g. zoom/Skype” and “Everyone should stay 2 m. (6 ft.) apart” (β = 16, p < .0050). Compliance of "Sensitive Interpreters" (n = 249) may be improved by messages such as, “Only people who are 60 and over are to be allowed to buy groceries during first 2 h from opening” and by using the media to publicize the official health policy (β = 8; p < .005).

**Conclusions:**

Mindset-assignment reflects how people think rather than their religious affiliation. A personal viewpoint identifier was developed to predict mindset-assignment and enable health authorities to enhance compliance through mindset-tailored messages for members of each mindset segment. We recommend that health authorities and policy makers consider these different personality types, which range across religious minorities and emphasize the messages that each type responds to in developing and implementing a communication plan to improve physical distancing as an important public health measure.

## Background

Physical distancing effectively reduces infection rates of COVID-19 [1, 2]. Physical distancing refers to maintaining physical distancing or separation to reduce close contact between people [3]. Practices of physical distancing include preventing assemblies of people in community settings and closure of schools, gyms, bars, and restaurants [4, 5]. Governments across the globe are implementing various practices of physical distancing as well as measures of hygiene and mandatory mask wearing [6]. The availability of a vaccine, however, does not ensure its uptake, thus, physical distancing is expected to remain the primary intervention to reduce the transmission of COVID-19 [7, 8]. Health authorities across countries invest efforts to implement the physical distancing policy by education, persuasion, legislation, coercion, and incentives [3, 9].

Despite these efforts and although physical distancing is vital in fighting the COVID-19, pandemic, compliance with physical distancing across religious minorities in Israel, has been shown to be relatively poorer than in the majority population, resulting in a high COVID-19 infection rate [10–17]. In Israel, although the Jewish ultra-Orthodox comprise 12.6 of the population, 40–60% of all coronavirus patients at four major hospitals were Jewish ultra-Orthodox and although the Arab population comprises 21% of the population, 33% of all coronavirus patients at four major hospitals were Arab [14, 18]. Studies highlight the importance of communication in promoting voluntary compliance with physical distancing, but studies on specific types of messaging are scant [19–22]. People may comply better with physical distancing if messages are crafted to complement voluntary behavior [19]. Echoing the social representation theory, researchers stressed the need to design culturally adapted messages calling for physical distancing, especially since the perception of risk has cultural roots [23, 24]. Consistent with the social representation theory, messages that reflect the shared reality of group members of each religious culture yield higher willingness to comply (hereafter: WTC) [12, 23].

Previous studies claimed that ‘culturally adapted’ messages may promote compliance across distinct religious cultural groups [24–26]. Policymakers were called upon to consider the unique characteristics, needs, culture and behaviors, of religious minorities in designing communication messages publicizing official guidelines to prevent the spread of the coronavirus, since failure to contain it among religious minorities will result in its spread to all other sectors of society [12, 14]. This study aimed at closing the gap in the state-of-the-art testing which specific messages drive willingness to comply with physical distancing across religious minorities.

WTC, an attitude, was found to be strongly related to compliance behavior [27]. Thus, identifying messages that drive WTC with physical distancing is essential for health authorities to effectively communicate the policy and achieve higher voluntary compliance across religious cultures [19, 25–28]. It is important to understand which messages drive willingness to comply with physical distancing, which messages have no effect, and which messages adversely affect WTC with physical distancing. This study explores these questions among religious minorities in Israel during the first wave of the COVID-19 from March till June 2020. Although the demographic composition in Israel differs from that of other countries, the strategy we present for designing messages that drive compliance with physical distancing can be applied in other countries. Following are characterizations of religious minorities in Israel.

In Israel, the ultra-Orthodox Jewish community and the Arab population (Muslim and Christian) are the most prominent and well-defined minority groups [29–32]. Since 1995, the Israeli Central Bureau of Statistics has been distinguishing between populations on the basis of two categories: “population group” and “religion.” There are three population groups: (1) Jews, who constitute 75% of the population; Arab Israelis, who account for 21% of the population, and others, 4% of the population which included non-Arab Christians, Buddhists, Hindus, Samaritans, and Bahá’ís. The Arab Israeli population accounted for 1.8 M in 2015 and comprises 84% Muslims, 8% Druze, and 8% Christians. Bedouins account for 16% of the Muslim population [33]. Bedouin communities are located in remote areas in Israel with poor access to healthcare and also require communication messages that consider the Bedouin unique cultural attributes in order to reduce infection rates [14].

Since during the first wave of the COVID-19 pandemic in Israel, the infection rate among the Jewish ultra-Orthodox and the Arabs was almost three times higher than expected, considering their percentage in the population, media campaign efforts were made in both the Israeli Arab population and the Jewish ultra-Orthodox population to distribute culturally relevant messages [34, 35]. Compliance with physical distancing, however, was poor and was attributed to unique cultural and behavioral attributes, such as large families, densely knit neighborhoods, a collectivist ethos, and adherence only to instructions given by the community's religious leadership [12, 14]. Furthermore, although these communities traditionally did not use the internet, in 2018–2019 internet used grew from 28 to 52% and due to COVID-19, the health ministry and academic colleges allocated computers to teachers and students, raising use to 66% [35]. Thus, although a major part of these populations is informed about guidelines of physical distancing, compliance with guidelines has been low.

Immense efforts were invested in creating effective communication channels and convincing the ultra-Orthodox leadership to mandate physical distancing in their communities, but compliance among some groups of these religious minorities is lower than in the general population [14]. While the general education system was under quarantine, some ultra-Orthodox educational institutions continued to operate. Although synagogues and mosques were instructed to shut down, a few religious leaders permitted their followers to continue assembling at their places of worship; subsequently, clusters of COVID-19 infections were identified among these worshippers [14].

Empirical studies testing the effect of messaging on the public's WTC are scant [3]. This research project seeks to start closing the gap in the state-of-the-art testing of the power of messaging in promoting WTC with physical distancing during the first wave of the COVID-19 pandemic across religious cultures. This research responds to previous calls to develop communication messaging models that incorporate creative strategies to tailor messages to diverse audiences [36–38]. This study examines which messages are strong drivers of WTC with physical distancing and which are neutral messages evoking weak responses across religious cultures. WTC with physical distancing was found to depend on: the perceived risk of the virus, the perceived benefits of physical distancing, and trust in the agent communicating the physical distancing policy [15, 38–43].

Furthermore, the influence of messages on WTC with physical distancing may depend, in part, on the extent to which people “identify” with the different messages [38]. Communication messaging may carry a different appeal to an unspecified group of people who show a similar pattern of responses to specific messages on physical distancing [38]. Also, the similarity in responses to a set of messages may lead to the emergence of profoundly different groups, so-called ‘mindsets. That is, individuals may differ from each other in many other ways but share a common way of reacting to the messaging on physical distancing. This experimental design explores the effectiveness of messaging to drive compliance with physical distancing across mindsets among religious minorities in Israel. The exploratory research questions are: What patterns of response are there to different components of the messages? Do these patterns differ by religious grouping?

## Methods

### Ethics

This study is part of a multi-national research project on compliance with physical distancing during the first wave of COVID-19 in Canada, the US, Hungary, Italy, Turkey, England, Australia and Israel. The study protocol was approved by the Ryerson University Research Ethics Board (#2020-149). Before participating in this online study, participants stated their agreement to participation and publication of the study results**.** Participants were informed that participation is anonymous and confidential, and that they can stop their participation at any time.

### Sample

The target population was Israeli residents ages 18 and over, from the Jewish secular population and the main religious culture groups: Jewish ultra-Orthodox, Muslims, Christians, Bedouins and Druze. Respondents were 896 Israelis from the different religious cultural groups (226 secular Jews, 218 Muslims, 94 Druze, 96 Christians, 168 Orthodox Jews, and 94 Bedouins). The sample comprised 371 females and 525 males, aged 18–75. Since our objective was to develop a model of messaging for each respondent, the question of sample size devolved into a question of the number of respondents needed before the average model, across respondents, becomes stable [44]. Whereas sociologists study behaviors of large groups of people and deal with the percent of people who achieve a given score, experimental psychologists deal with individual behavior, focusing on the magnitude of a response and looking at means, and the stability of the mean as a predictor of the performance of the dependent variable. In Mind-Genomics®, since results are based upon the average rating assigned to a message, the size of the sample is not a question of the stability of the average rating but rather the stability of the utilities that the model averaged across the different respondents. Data on utilities from several conjoint measurement samples confirm previous observations on base size studies and indicates that much of the information can be obtained with lower bases than the typical base size, and the same conclusions can be drawn with base sizes of around 50 respondents per subgroup in the population [45]. Thus, the size of the total sample and of the subgroups is consistent with the suggested sample size in conjoint analysis studies, particularly when aiming at stability of coefficients rather than stability of means [44]. Table 1 presents the sample demographics.

**Table 1:** Sample demographics

Total	896
Male	525
Female	371
Secular Jews	226
Muslims	218
Druze	94
Christians	96
Orthodox Jews	168
Bedouins	94
Age 13–17	19
Age 18–24	141
Age 25–34	194
Age 35–44	197
Age 45–54	203
Age 55–64	111
Age 64 +	31

### Procedure

We utilized an experimental design in which we allocated participants to different groups using repeated measures, where the same participants took part in each condition of each of the independent variables (within groups, or within-subjects design). In this experimental design, participants rated a series of different combinations of messages with the same rating question. This way, participants did not complete “repetitions” or “parallel measures” but were repeatedly exposed to the same question in relation to different aspects of physical distancing. To control the results, we alternated the order by which participants performed in different conditions of an experiment. Compared to typical observational studies, this experimental design enables higher variation, randomization, analysis of co-variance, and control [46]. Since our reality is complex and encompasses many stimuli that may interact with one another, we utilized a conjoint based experimental design, Mind-Genomics**®**, well acknowledged in academia and industry for testing the power of messages and uncovering mindsets [44, 47–50]. Mind-Genomics**®** has been used to test the power of messages in a great variety of topics, from meat analogues through distance learning in higher education and people’s reactions to physical distancing measures. We applied Mind-Genomics® to test the preferences of people regarding messages that drive their WTC with physical distancing [51]. Data collection began on May 1st, 2020 and lasted one month. Numerous messages were tested (5000 +) with no limitation of degrees of freedom [52]. A digital link for this online study was distributed through social networks of representatives of community agencies in a snowball sampling. Among Bedouins, who are generally not exposed to social networks, research assistants personally assisted respondents to use an iPad to fill out the survey.

### Instrument

**Fig. 1:**
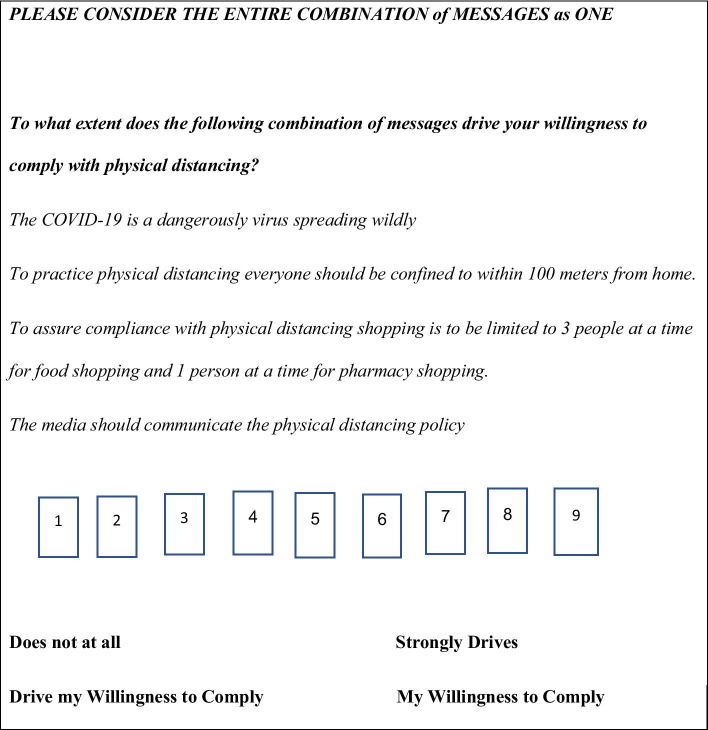
An illustration of a presentation of messages and the rating question

The dependent variable is ‘WTC with *physical distancing*’. As typical in conjoint-analysis messages fall into four categories, each acknowledged as a driver of willingness to comply with physical distancing. Each category contained four messages, limited to one from each category, altogether sixteen different messages. Messages were created based on elements we identified in a thorough literature search regarding independent variables and were previously published [38]. Each participant received 24 combinations of messages, with only one message allowed from each category [40–43, 51, 53, 54], and were instructed to rate the combination as a unity [51, 54]. The rating question was: "To what extent does the following combination of messages drive your willingness to comply with physical distancing?" The rating question appeared on each screen above the combination of messages. The rating scale ranged on a scale of 1 (Does not at all drive my willingness to comply with physical distancing) to 9 (Strongly drives my willingness to comply with physical distancing. Figure 1 illustrates one out of 24 presentations of messages that respondents were asked to rate.

The test stimuli of combinations of messages were dictated by a well-crafted mathematical method called an ‘experimental design' which structures the 24 combinations to ensure the statistical independence of the predictor variables for subsequent regression at the level of the individual respondent and at the level of the group [51, 53, 54]. The combinations generated a compound message pulling in different directions which forced the respondents to evaluate the combination using their intuition, thereby reducing typical biases of surveys [51]. Reliability of the instrument was tested by split halves comparing data for the total sample with data for half of the sample (0.70; 0.76). Table 2 presents the study instrument.

**Table 2:** Messages by categories of known drivers of willingness to comply

Category 1: Perceived risk of COVID 19	
A1	Very dangerous, wildly infectious
A2	Not dangerous; media exaggerates the strain of influenza
A3	Not dangerous, but it is all they talk about in the news
A4	Experts are advising but the government is reactive
Category 2: Practices of physical distancing	
B1	Everyone should work and study only from home
B2	Everyone should keep a distance of 2 m from each other
B3	Everyone should be Confined to within a 100 m distance from the house
B4	Everyone should wear a mask everywhere we go
Category 3: Ways to ensure physical distancing	
C1	A compulsory lockdown
C2	Leave only for grocery shopping (3 at a time) or pharmacy (1 at a time)
C3	Designated volunteers do shopping for elders and disabled
C4	Only 60 and older shop in the first two hours after opening
Category 4: The agent communicating the physical distancing policy	
D1	National government
D2	Home Front Command
D3	Religious leaders
D4	The media

### Data analysis

The experimental design enabled the deconstruction of responses to the messages by ordinary least-squares regression (OLS) [53, 54]. We created 896 models for WTC using OLS, one for each respondent, each with both an additive constant and 16 coefficients, one coefficient for each message. The additive constant is a purely estimated parameter, the intercept in a linear equation that may be interpreted as the predisposition of the respondent group to agree to a set of messages in the absence of any specific message. High additive constants (60 +) represent groups of people who are likely to agree with what they are presented. Messages with low values, or negative values, detract from the high level of basic agreement with messaging. Low additive constants (< 35) represent groups of people who are unlikely to agree with what they are presented. The specific messages drive agreement, not the general proclivity to agree.

We performed OLS to generate individual level equations for each respondent relating to the presence/absence of the sixteen messages. The OLS coefficient is the conditional probability that the specific message adds to the perceived importance of the additive constant for WTC. A coefficient of six or higher is statistically significant, given the standard error of about 4 for the coefficient. A higher coefficient means higher WTC. OLS was run for the total panel, for each religion culture and for key subgroups (gender, age), incorporating all relevant data into one regression model for the sample. The response to these combinations, uncovered by OLS, reveals the part-worth contribution of each message to WTC with judgment bias reduced [54]. Since the self-ratings of respondents are not calibrated, following OLS the rating was transformed to a categorical variable (1–6 = 0; 7–9 = 1), enabling the reduction of variability and crystallization of the strongest drivers of WTC.

Next, we analyzed response patterns to each message, using *k*-means clustering algorithm with 1-Pearsons’s R distance measure [53]. Fundamental groups, so-called mindset-segments, emerged. ANOVA and Post Hoc tests indicated that mindset models were significant. These mindsets highlight the different specifics of communication that drives WTC for each religion culture. The pattern of positive high coefficients across different mindsets guided the assignment of respondents to mindset.

Last, to translate the knowledge derived in this study to policy implementation, we developed a prediction tool, the Personal Viewpoint Identifier (PVI). The PVI tool is a method by which health authorities may assign a person or group in the population to a mindset. The PVI is created using the Monte-Carlo simulation 100 times, for validation, by identifying messages with the highest differences among their coefficients, thereby strongly discriminating among mindsets [51]. The PVI is based on the summary data, converting the strong distinguishing messages to binary questions (agree or disagree). The PVI becomes a short binary questionnaire, enabling health authorities to assign individuals quickly and easily into a mindset.

## Results

The response rate for the on-line sample was a high response rate of 66%. Out of 1,204 people who started the online study, 797 completed it. The experimental design enabled the deconstruction of responses to the messages by ordinary least square (OLS) regression [15, 39]. We created models for WTC using OLS, one model for each respondent, each with an additive constant and 16 coefficients (i.e., one coefficient for each message). The additive constant is an estimated parameter representing the intercept in a linear equation that may be interpreted as the predisposition of the respondent group to agree to a set of messages in the absence of any specific message. The response to each combination of messages, the coefficient of the OLS, reveals the importance that each respondent attributes to each message [42, 43].

To highlight the best-performing messages and eliminate a high variability due to lack of calibration among respondents, we transformed the ratings to a binary scale. Ratings 4 and 5 (upper 40% of the scale) were transformed to 100, classified as positive outcomes; ratings below 4 (lower 60% of the scale) were transformed to 0, classified as negative outcomes. OLS analysis was performed to create an individual-level regression model for each respondent. This type of individual regression approach has been widely used in conjoint analysis studies [52]. The OLS model was written as follows:$\widehat{Y}={\beta }_{0}+{\beta }_{1}{X}_{1}+{\beta }_{2}{X}_{2}+\cdots +{\beta }_{p}{X}_{p}$, where $\widehat{Y}$ is the predicted or expected value of WTC (here, the transformed, binarized ratings),${X}_{1}$ through ${X}_{p}$ are $p$ distinct independent or predictor variables, ${\beta }_{0}$ is the value of $Y$ when all of the independent variables (${X}_{1}$ through ${X}_{p}$) are equal to zero, and ${\beta }_{1}$ through ${\beta }_{p}$ are the estimated regression coefficients. The OLS coefficient is the conditional probability that the specific message adds to the perceived importance of the additive constant for physical distancing. OLS was run for the entire panel, incorporating all relevant data into one regression model for the sample. The regression model, estimated at the level of each respondent, is appropriate because of the permuted design.

To simplify the analysis, we presented only messages with positive regression coefficients, driving WTC with physical distancing. Negative regression coefficients mean either that the element is neutral (irrelevant for WTC) or counterproductive, driving non-compliance. Regression coefficients for the models relate to the presence/absence of the elements to the rating of disagree/agree, after binary transformation. (*denotes significant, positive model parameters (*p* < 0.05)). Table 3 shows that whereas for each culture a different message was the strongest driver of WTC, the t and p values of the OLS regression indicated that the differences among coefficients were not significant and there were no clear differences by gender, by age, or by self-defined religion cultural group.

**Table 3:** Coefficients of drivers of willingness to comply with physical distancing

	Total	Jew Secular	Jew Orthodox	Muslim	Christian	Druze	Bedouin
**Additive constant**	**39**	**30**	**38**	**50**	**42**	**40**	**37**
Situation: dangerous virus spreading wildly	1	0	1	1	1	2	4
Situation: health experts suggest what to do but the government has been reactive rather than proactive to the pandemic	2	1	1	1	5	0	7
Situation: all the news seems to be about the COVID-19 virus	1	− 1	2	0	3	2	7
Situation: media exaggerates new strain of influenza…people are panicking	1	2	3	0	1	1	1
Physical distancing: everyone should socialize and work only from home on internet, e.g. zoom/Skype	0	− 1	0	− 2	0	3	1
Physical distancing: everyone should stay 2 m (6 ft.) apart	− 1	− 2	1	− 3	− 2	1	0
Physical distancing: Everyone should wear a mask everywhere	− 2	− 2	0	− 3	1	− 3	− 2
Physical distancing: everyone should be confined to within 100 m (300 ft.) of home	− 1	− 4	1	− 5	0	4	0
Who communicates the policy: home front command	2	7	2	− 1	− 2	− 3	2
Who communicates the policy: state government	0	3	2	− 1	− 2	− 5	1
Compliance policy: only 60 and over allowed to buy groceries during first 2 h of the store day	0	5	− 2	− 1	− 3	− 1	− 2
Who communicates the policy: media	1	5	2	− 3	− 2	− 3	3
Who communicates the policy: religious leaders	0	3	1	− 1	− 2	− 4	3
Compliance policy: designated young volunteer for priority shopping… for elderly & disabled	− 1	1	2	− 3	− 2	− 2	2
Compliance policy: food (3 people at a time) gas (attendant dispenses)…pharmacy (1 person at a time)	0	2	− 1	− 1	− 3	− 2	3
Compliance policy: military lockdown	0	2	− 2	1	− 3	− 5	0

*K-*means clustering was applied on the 16 coefficients to create clusters. These clusters represent mindsets because they suggest what is important to the respondent. Mindsets emerge from the pattern responses to the specific, relevant messages, not from stated attitudes. Following mathematical clustering, the equation for each subgroup was estimated using all data from the appropriate group. Analysis of variance and post hoc tests indicate whether the distinct mindset models were significant, highlighting the different messages that impact WTC with physical distancing for each mindset. The pattern of positive high coefficients across different mindsets guided the assignment of respondents to a mindset. Significant differences emerged when the respondents were clustered by the pattern of their responses to the individual messages, the mindsets. The data suggest three distinct groups, emerging from the k-means clustering [54]. Mindsets are: "Pandemic Observers", who pay close attention to the news; "Obedient Followers", who expect to be told EXACTLY what to do; and "Sensitive Interpreters" who are attentive to what the government decides. The names of the mindsets were determined by the dominant messages in each mindset. These three mindsets transcend religious culture, age, and gender. Table 4 presents the additive constant, coefficients, p values, and post hoc results.

**Table 4:** Mindsets Emerging from Similarity in Patterns of Responses to Messages

	Total	Mindset 1	Mindset 2	Mindset 3
**Additive constant**	**40**	**39**	**44**	**35**
**Mindset 1—"pandemic observer" (focus on risk and pay attention to the news)**				
Perceived risk: dangerous virus spreading wildly	1	14^c^	− 12^a^	0^b^
Perceived risk: health experts suggest what to do but the government is reactive rather than proactive	2	14^c^	− 11^a^	1^b^
Perceived risk: all the news seems to be about the covid-19 virus	1	13^c^	− 13^a^	2^b^
Perceived risk: media exaggerates the strain of influenza…people are panicking	1	13^c^	− 11^a^	0^b^
**Mindset 2—"obedient follower" (focus on practices of physical distancing and expect to be told EXACTLY what to do)**				
Practice physical distancing: Everyone should socialize and work only from home on internet, e.g. zoom/Skype	0	− 9^a^	− 5^b^	**16**^**c**^
Practice physical distancing: Everyone should stay 2 m (6 ft.) apart	− 1	− 12^a^	− 6^b^	**16**^**c**^
Practice physical distancing: Everyone should wear a mask everywhere	− 2	− 12^a^	− 4^b^	**11**^**c**^
Practice physical distancing: Everyone should be confined to within 100 m (300 ft.) of home	− 2	− 13^a^	− 7^b^	**17**^**c**^
Who communicates: Home Front Command	2	− 5^a^	10^c^	0^b^
Who communicates: State Government	0	− 5^a^	7^c^	− 1^b^
**Mindset 3—"sensitive interpreter" (focuses on ensuring compliance and on the communicator, listening to WHAT the GOVERNMENT IS DOING)**				
Ensuring compliance: Only 60 and over allowed to buy groceries during first 2 h of the store day	0	2^b^	**8**^**c**^	− 11^a^
Who communicates the policy: media	1	− 4^a^	**8**^**c**^	0^b^
Who communicates the policy: religious leaders	0	− 7^a^	**6**^**c**^	1^b^
Compliance policy: Designated young volunteer for priority shopping… for elderly & disabled	− 1	3^b^	**6**^**b**^	− 12^a^
Compliance policy: food (3 people at a time) gas (attendant dispenses)…pharmacy (1 person at a time)	0	2^b^	**6**^**b**^	− 10^a^

All elements showed significant (p < 0.01) differences among mind-sets. Letters denote significant differences among mind-sets based on Tukey post hoc test

Since the three mindsets are distributed across religious cultures, gender, and age groups as shown by the distribution of the three emerging mindsets, a PVI is required to identify the belonging of individuals in the population to a mindset. Table 5 presents the PVI using six strong messages that distinguish among mindsets.

**Table 5:** Personal viewpoint identifier for mindset-assignment

Element	Answer
Practice physical distancing: everyone stays 2 m (6 ft.) Apart	○ Agree
	○ Disagree
Perceived risk: all the news seems to be about the covid-19 virus	○ Agree
	○ Disagree
Practice physical distancing: socialize and work only from home on internet, e.g. zoom/skype	○ Agree
	○ Disagree
Ensuring compliance: only 60 and over allowed to buy groceries during first 2 h of the store day	○ Agree
	○ Disagree
Practice physical distancing: confined to within 100 m (300 ft.) Of home	○ Agree
	○ Disagree
Perceived risk: dangerous virus spreading wildly	○ Agree
	○ Disagree

## Discussion

This research starts to close the gap in the state-of-the-art testing the power of numerous combinations of messages that drive WTC with physical distancing across religious cultures in Israel. This research has theoretical, methodological, and practical contributions. Theoretically, its findings contradict the social representation theory. In the case of COVID-19, only the perceived risk of the virus and specific dictated practices of physical distancing drove WTC. The collective social representation didn't provide a strong structure for driving WTC with physical distancing across religious cultures. Respondents from the same religious culture didn't agree on the same messages but rather, showed differential sensitivities, yielding a non-significant impact of most messages. Thus, contrary to the belief that conventional messaging may not be effective among religious minorities, in the context of a health crisis, people make decisions based on their perceived risk, practices of physical distancing, the agency communicating the messages, and ways the guidelines are enforced [38].

The contradiction between our findings and the theory of social representation may be explained by the concept of ‘cognitive polyphasia' that members of distinct religious cultures exhibit in light of extreme events at the national level, leading members to employ a number of different social representations that pertain to the same topic [55, 56]. Even within one religious culture group, there may be different sources of information about the same topic, generating a variety of ways that people processed the information and only then connected it to the social context of the culture. Members of the six religious cultures may have obtained different information because of who they are as a group (i.e., lack of information, little exposure to mass communication, and to networks), and because of their individual experiences in the situation, (i.e., being infected, quarantined or hospitalized), illustrating 'discursive polyphasia', i.e., the ability to hold a variety of different and sometimes inconsistent ideas about a subject at the same time [56]. Thus, this notion of discursive polyphasia may account for the three mindsets emerging *across* religious cultures rather than *within religious cultures* [56].

Methodologically, this study used a patented methodology of conjoint-based experimental design, overcoming the typical biases of surveys and simultaneously testing various messages that were crafted to reflect the complexity in reality impacting an individual’s WTC with physical distancing as a way to explore the strategy of communication by mindset-assignment. Practically, although each person has different needs and sensitivities that may challenge effective messaging in a health crisis, tailoring the information to diverse religious populations may be more effective when based on mindset-assignment as we demonstrated in this study.

Data suggest three distinct mindsets regarding agreement with specifics about compliance with physical distancing. Findings also suggest that health authorities communicate all their messages through the media and not through religious clergy or politicians. In contrast to the collective social representation theory, the mindsets transcended religion and represent all religious cultures, albeit in different proportions. Members of mindset 1 are affected by messages stressing the risk of the virus. They are "Pandemic Observers", who pay close attention to the news; these constitute 38% of the respondents, the largest group. Members of mindset 2, are affected by specific practices of physical distancing. They are "Obedient Followers", expecting to be told EXACTLY what to do; these constitute 31% of the respondents. Members of mindset 3, also 31% of the respondents, are affected by messages on the ways to ensure compliance and on the communicator of the policy; these are "Sensitive Interpreters" who are attentive to what the government decides. Each mindset was comprised of people from all religious cultures. The hypothesis was not supported.

Mindset-assignment did not reflect affiliation with a certain religious culture, but rather reflected differences in the way people from any religious culture think. Attitudes and perceptions regarding health precautions in COVID-19, including physical distancing, were found to be stable [57]. We believe that possible changes in attitudes towards physical distancing as well as changes in the willingness to comply with physical distancing are "random errors" that do not affect either the revealed mindsets or the assignment into mindsets.

Findings echo a previous study suggesting that a unified message for everyone regarding law-enforceable behaviors during a crisis is ineffective [38]. Members of each mindset *cannot be easily identified just by knowing who they ARE by means of demographic data*. To shape public behaviors in a pandemic, epidemiologists must communicate using messaging based on the proclivities of the mindsets of their audience and on how the messaging may influence public WTC with physical distancing across religious cultures. Our PVI enables public health authorities to identify the mindset of each group or person in the population and use the most effective messages that drive WTC by the mindset-assignment.

### Practice implications

To enhance compliance with physical distancing, health policy makers and health authorities are called upon to consider the novel strategy of customizing messages by mindset assignment rather than by demographics. Policy makers are called upon to consider the proclivities and sensitivities of each of the three mindsets when properly implementing the policy of physical distancing. We suggest applying the prediction tool we developed based on mathematical clustering. This web-based user friendly tool entails six messages that strongly distinguish among mindsets. Individuals and goups may rate messages on a binary scale as part of a baseline repository. Authorities will be able to quickly identify the mindset of each individual or group and communicate through mindset-tailored specific messages that drive the WTC among members of each mindset. To customize messages on a large scale, individuals may be directed to a web-based page through their health maintenance organization and fill in the PVI questionnaire. Based on their ratings of the six mindset distinguishing messages in the PVI, they will each be assigned to one of the three mindsets, and health authorities will communicate mindset-tailored messages for individuals or groups**.** The use of the PVI prediction tool and communicating mindset-tailored messages may optimize the public's WTC with physical distancing with greater specificity, thereby achieving higher effectiveness in promoting compliance with physical/physical distancing.

### Study limitations

The pandemic is still unfolding. Study findings are based on data collected in the first wave of COVID-19, which surfaced in full force from March 2020 to late July 2020. Drivers of WTC with physical distancing may change across future waves. In addition, WTC may be affected by the exposure to messages or the priming effect of messages*.*

### Future studies

Future studies may test the effect of using the appropriate messaging by mindset on WTC. Future studies may also test the difference of exposure to messages and the role of priming effects of messages on compliance to physical distancing. Positive outcomes may create an impetus to further investigate the concept of ‘resonance’ and its role in explaining persuasive messages in health crises.

## Conclusions

Members of the three mindsets are dispersed across religious cultures. To drive WTC, epidemiologists are called upon to explore the strategy we presented here, to use the PVI to easily identify mindset belonging and design specific messages for each mindset when communicating the social-distancing policy. Although all religious minorities are collectivist minorities whose members follow the directives of their leadership, findings call upon health authorities to identify the individual mindsets and thus better tailor the messages for optimum compliance, and communicate messages through the media, not through religious clergy or politicians. During pandemics, the communication resources of governments become scarce. Effective messaging enables health authorities to allocate resources based on real, immediate, and relevant data to persuade religious cultural groups to comply with physical distancing. Employing effective messaging, health authorities will communicate with greater specificity and a higher likelihood of driving voluntary compliance with physical distancing across religious cultures [58].

## Data Availability

Upon acceptance authors agree to share data associated with this paper.
